# *Andrographis paniculata* decreases fatigue in patients with relapsing-remitting multiple sclerosis: a 12-month double-blind placebo-controlled pilot study

**DOI:** 10.1186/s12883-016-0595-2

**Published:** 2016-05-23

**Authors:** J. C. Bertoglio, M. Baumgartner, R. Palma, E. Ciampi, C. Carcamo, D. D. Cáceres, G. Acosta-Jamett, J. L. Hancke, R. A. Burgos

**Affiliations:** Instituto de Medicina, Universidad Austral de Chile, Valdivia, Chile; Departamento de Neurología, Hospital Regional de Valdivia, Valdivia, Chile; Centro de Esclerosis Multiple, Facultad de Medicina, Pontificia Universidad Católica, Santiago, Chile; Programa de Salud Ambiental, Escuela de Salud Pública, Facultad de Medicina, Universidad de Chile, Santiago, Chile; Instituto de Medicina Preventiva Veterinaria y Programa de Investigación Aplicada en Fauna Silvestre, Facultad de Ciencias Veterinarias, Universidad Austral de Chile, Valdivia, Chile; Instituto de Farmacología, Facultad de Ciencias Veterinarias, Universidad Austral de Chile, Valdivia, Chile

**Keywords:** *Andrographis paniculata*, Fatigue, Multiple sclerosis

## Abstract

**Background:**

*Andrographis paniculata* (*A. paniculata*)*,* a medicinal plant, has shown anti-inflammatory, neuroprotective and antifibrotic effects in animal models as well as clinical efficacy in different studies, including an anti-fatigue effect in autoimmune diseases such as rheumatoid arthritis. In multiple sclerosis (MS), fatigue is rated as one of the most common and disabling symptoms. In the present trial, we investigated the effect of *A. paniculata* on relapse rate and fatigue in relapsing-remitting MS (RRMS) patients receiving interferon beta.

**Methods:**

A randomised double-blind placebo-controlled trial assessed the effects of 170 mg of *A. paniculata* dried extract tablet b.i.d. p.o. on relapse rate and fatigue using the Fatigue Severity Scores (FSS) over 12 months in RRMS patients receiving interferon. The Expanded Disability Status Scale (EDSS) score, inflammatory parameters and radiological findings were also investigated. Twenty-five patients were enrolled, and twenty-two patients were ultimately analysed and randomised to the active or placebo group.

**Results:**

Patients treated with *A. paniculata* showed a significant reduction in their FSS score as compared to the placebo, equivalent to a 44 % reduction at 12 months. No statistically significant differences were observed for relapse rate, EDSS or inflammatory parameters, with a trend in reducing new lesions among the *A. paniculata* group. One patient in the *A. paniculata* group presented with a mild and transient skin rash, which was alleviated with anti-histamine treatment for three weeks.

**Conclusion:**

*A. paniculata* was well tolerated in patients and no changes in clinical parameters were observed. *A. paniculata* significantly reduces fatigue in patients with RRMS receiving interferon beta in comparison to placebo and only interferon beta treatment.

**Trial registration:**

*ClinicalTrials.gov* Identifier: NCT02280876; Trial registration date: 20.10.2014.

## Background

Multiple sclerosis (MS) is an autoimmune disease characterised by inflammation and neurodegeneration within the central nervous system (CNS). Its development seems to be multifactorial, with genetic predisposition influenced by environmental factors. It represents the main cause of non-traumatic neurological disability in the young adult population (20–40 years) [1].

Fatigue is defined as “*a subjective lack of physical or mental energy that is perceived by the individual or caregiver to interfere with usual and desired activities*” [1]. Fatigue is rated as one of the most common and disabling symptoms in MS and other autoimmune diseases. Its prevalence ranges from 65 to 97 % among MS patients, and it tends to seriously impair approximately one-third of them, with 55 % describing it as one of the worst symptoms they experience [2]. Fatigue has a significant impact on patients’ quality of life, affecting performance at work as well as their social and private lives [3]. Fatigue has also been associated with cognitive dysfunction [4, 5] and sleep disorders [6, 7]. Using functional magnetic resonance imaging, it has been proposed that a dysfunction of critical cortical areas contributes to the occurrence of central fatigue [8].

Despite many investigations, the pathophysiology underlying MS-related fatigue is not yet clear. Proposed mechanisms for fatigue include primary causes such as grey matter atrophy [9, 10], demyelination and axonal loss [11], functional cortical reorganisation [12], neuroendocrine deregulation [13] and immune system dysfunction and systemic inflammation [14]. Until now, no pharmacological treatment has shown marked efficacy in fatigue alleviation [2]. Recently, alfacalcidol treatment has shown promising effects in the reduction of fatigue in MS patients [15].

*Andrographis paniculata* (*A. paniculata*) is a native medicinal plant from Southeast Asia. *A. paniculata* extracts have been used in autoimmune diseases including rheumatoid arthritis and ulcerative colitis with some degree of success [16–19]. Many studies indicate that andrographolide, the main labdane diterpene present in *A. paniculata*, is responsible for the reduction of innate and adaptive immune responses [20–22]. Additionally, other studies have suggested that andrographolide might exert neuroprotective effects, that is, effects against damage induced by dopamine in mesencephalic neuron-glial cultures associated with a protective effect on inflammation-mediated neurodegeneration [23], oxidative stress induced by nicotine in the brain [24] and cerebral ischemia [25].

Animal studies have shown that andrographolide ameliorates the symptoms of experimental autoimmune encephalomyelitis (EAE) by interfering with the inductive phase of EAE [22], which is accomplished by inhibiting T cell and antibody responses directed to myelin antigens and interference with the maturation of dendritic cells (DCs) and their ability to present antigens to T cells. Furthermore, in *mdx* mice that shows muscular fibrosis and physical weakness, andrographolide induces an increase in exercise endurance [26]. In a previous placebo-controlled clinical pilot study in patients with rheumatoid arthritis, *A. paniculata* was able to significantly reduce fatigue after 90 days of treatment versus the placebo [16]. All this background information suggests a potential use for andrographolide and/or *A. paniculata* in MS patients.

On the other hand, the active compounds of *A. paniculata* have shown low toxicity, as reported by different clinical studies. The most common adverse event presented in some isolated cases is the development of mild skin rash [19], probably associated with andrographolide or its metabolites. It disappears upon withdrawal of the drug.

In the present pilot study, we show evidence that a purified extract of *A. paniculata* is able to significantly reduce fatigue in patients with RRMS receiving interferon at 12 months.

## Methods

### Trial design

This pilot study was a randomised, double-blind, placebo-controlled, single-site study conducted at the Regional Hospital of Valdivia, Neurology Department, Faculty of Medicine of the Universidad Austral de Chile to evaluate the effect of *A. paniculata* in RRMS patients treated with interferon beta.

The trial was conducted in accordance with the International Conference on Harmonisation Guidelines for Good Clinical Practice and Applicable Local Regulations, as well as the Declaration of Helsinki. The Chilean Ministry of Health, Ethical Scientific Committee of the Health Service of Valdivia and the local Ethics Committee approved the clinical protocol. All participants signed informed consent forms. The trial was registered on ClinicalTrials.gov: NCT02280876. The diagnostic criteria for MS were in accordance with MacDonald 2010 [27]. As far as possible, the CONSORT 2010 guidelines for reporting parallel group randomised trials were followed [28].

### Participant

Inclusion criteria: (a) age between 18 and 50 years (inclusive), (b) relapsing-remitting MS, (c) Expanded Disability Status Scale (EDSS) score ≤ 6.0 (due to public health regulations, patients with EDSS >6 are not allowed to begin any disease-modifying drug), (d) at least one relapse documented in the previous two years, (e) treatment with interferon beta-1a [intramuscular (Avonex®) once a week or subcutaneous (Rebif®) three times a week].

Exclusion criteria: (a) previous immunosuppressive treatment (e.g., rituximab, mitoxantrone, cyclophosphamide), (b) pregnancy, breastfeeding or absence of effective contraception, (e) uncontrolled systemic diseases or a life-threatening and/or unstable clinical condition and/or alcohol or drug abuse, and (f) psychiatric disorders that could affect treatment compliance.

Withdrawal causes from study included: (a) relapse that needed treatment with corticosteroids; (b) necessity of second line drugs, immunosuppressive treatment or plasmapheresis; (c) pregnancy; (d) serious systemic disease; and (e) voluntary abandonment. If a mild relapse (i.e., only sensory symptoms) occurred during the study, the subject was offered the possibility to re-consent and continue with the protocol.

To assess similarities between the baseline clinical characteristics of the patients treated with *A. paniculata* and the placebo group, a Mann–Whitney *U* test was carried out. No statistical differences existed between the groups (Table 1).

**Table 1:** Baseline clinical and radiological characteristics of patients after randomisation in *A. paniculata* and placebo treatment groups

Parameter		*A. paniculata n* = 13					Placebo *n* = 11				
		Mean	SD	min	max		Mean	SD	min	max	*p-value*
Age		35.09	11.79	15	47		38.70	10.65	22	51	*0.3066*
Sex (w/m)		9/4					7/4				*0.5250*
Disease duration prior to study (year)		3.62	4.56	0	16		6.00	8.20	0	24	0.8067
Relapse (2 years)		1.73	1.27	1	5		1.46	0.52	1	2	0.9236
EDSS		2.64	1.29	1	5		2.08	1.66	0	6	0.1663
FSS		4,15	1,88	1	6.4		3.76	1.68	1	5.5	0.3990
*Gd* lesion number	0					0					
Interferon-beta 1a im.(Avonex® 30 mcg)	7					5					
Interferon-beta 1a sc.(Rebif® 44 mcg)	6					6					

Patients were assigned to two neurologists who were blind to the patients’ treatment. One physician was responsible for the clinical aspects of patient care, including the evaluation and management of adverse events and acute relapses. The other physician rated patients EDSS and FSS scores, and was also blind to patients symptomatology.

Patients were examined every three months for a period of 12 consecutive months. In case of a suspected relapse (new or worsening neurologic symptoms not associated with fever or infection that lasted at least 24 h and were accompanied by new objective neurologic signs), the patient would be referred to the rater neurologist, who would confirm or dismiss the relapse. At the discretion of the treating neurologist, relapses would be treated with intravenous methylprednisolone at a dose of 1000 mg per day for three consecutive days, and patients would be withdrawn.

### Intervention

Eligible patients were randomised to orally receive one tablet containing 170 mg of *A. paniculata* purified extract (total andrographolides: 85 mg per tablet) or placebo every 12 h for 12 consecutive months, which was estimated according to previous clinical trials [16]. Both formulations comply with “Good Manufacturing Practice” and the norms N° 04/2009 from the Institute of Public Health of Chile (IPH-Chile). The product was authorised and registered as an Investigational New Drug (IND), dated 07-30-2013 for IPH-Chile and numbered 2390. The herbal medicine intervention used in this trial was a highly purified composition of standardised dried extract of *A. paniculata* (Burm. f.) Wall. ex Nees (Acanthaceae). The extract of *A. paniculata* was provided by Innobioscience Spa, Chile and manufactured by EUROlab® laboratories Chile, in blister packs (10 units each) containing film-coated circular tablets with soft convex surfaces, and blue colour weighing 650 mg in carton boxes for a month of treatment (60 units). The placebo was manufactured with the same characteristics and packaging as the active drug, containing only excipients.

The drugs were kept according to the instructions of the manufacturer and separated from the hospital’s normal stocks. The extract was obtained from leaves and aerial parts of *A. paniculata* that had been kindly provided by InnoBioscience, LLC (Miami, USA). The active product tested was a standardised dried extract of *A. paniculata*. The extraction procedure was performed with alcohol (75 % ethanol), and the ratio of herbal drug to extract was 10:1. A botanist identified the plant. The batch number of *A. paniculata* extract used in this study was PAR-130101-7. A voucher specimen was conserved (no. 20050520) and kept at InnoBioscience Spa (Miami, USA). The posology was established according to previous clinical trials [16, 29]. The percentages of quantified chemical constituents per tablet was as follows: 85 mg of total andrographolides (50 % w/w), which were comprised of approximately 3 % w/w of 14-deoxyandrographolide and 0.2 % w/w of neoandrographolide.

A chemical fingerprint for the extract of *A. paniculata* was performed. The method of analysis was as follows: The compounds were extracted with acetone (4:1) and then analysed by high-performance liquid chromatography (HPLC) using a reverse-phase RP-C18 licrospher column (4 × 125 mm). The mobile phase was 26 % acetonitrile and 0.5 % phosphoric acid, eluted at a rate of 1.1 ml/min, using a wavelength of 228 nm according to Burgos et al. [30]. The product sample is also kept at the Laboratory of Pharmacology, University Austral of Chile. The following reference standards were used: andrographolide (98 %) purchased from Sigma (St. Louis, MO) and 14-deoxyandrographolide (90 %) and neoandrographolide (90 %) supplied by Indena SpA (Milano, Italy). The purity of these reference standards was assumed as provided by the suppliers. The placebo tablets used in this trial were identical in size (lactose powder filling) and colour (with food colouring) to the *A. paniculata* tablets.

### Outcomes

The primary outcome of the trial was to evaluate the efficacy of *A. paniculata* on fatigue in a group of RRMS patients receiving interferon beta in a period of 12 months. Fatigue was measured by the FSS score using a self-rating questionnaire, a reliable and validated nine-item statement concerning respondents’ fatigue, including how fatigue affects motivation; exercise; physical functioning; carrying out duties; and work, family or social life. The scale was a seven-point Likert scale, where 1 = ‘strongly disagree’ and 7 = ‘strongly agree’. Sum responses are divided by the number of items for scale score, with a range of 1 to 7, where higher scores indicate more severe fatigue, with a mean value of 2.3 (SD ± 0.7) in normal healthy adults [31].

Secondary outcomes including other clinical parameters included the relapse rate assessed by the treating neurologist and confirmed by the EDSS rater, which was defined as the appearance of new symptoms or the return of old symptoms for a period of 24 h or more in the absence of a change in core body temperature or infection. In addition, safety, tolerability and adverse events, including a symptom diary where patients could record any untoward symptom, were recorded. Magnetic resonances, including T2-FLAIR and T1 pre- and post-gadolinium (0.1 mmol/Kg), were also performed at baseline and after 12 months of therapy, and the number of T2- and gadolinium-enhancing lesions were assessed at these time points. Plasmatic inflammatory activity parameters (cytokines) in groups treated with *A. paniculata* and placebo were also measured.

Treating neurologists were instructed to report any adverse experience, including duration, severity, action taken, relationship to the study drug and outcome of the event. Patients were also encouraged to report any adverse event in their symptom diary.

### Randomisation, allocation concealment and blinding

All appointments for the 25 participants were arranged at the Neurology Department of Regional Hospital, Valdivia, Chile. Twenty-four patients met the inclusion criteria and were randomised into active or placebo groups based on the order of their chosen date and the arrival time for their clinical assessment. These were assigned to each treatment through a random sampling design using balanced blocks. A total of 12 placebo and 13 active patients were allocated to each group, according to the study statistician’s computer-generated allocation sequence. Participants were not informed of their group assignment code. Patient assignments were generated with the statistical and epidemiology data analysis software EPIDAT 3.1 [32].

The study physicians did not share their own examination results, did not handle the study products and did not know the assigned treatment. Two envelopes contained each individual’s treatment assignment; one set was for the Neurology Department to keep for emergency care and the other was kept with the Principal Investigator. The two envelopes remained sealed until data analysis. The appearance of the test product and placebo-coated tablets was identical, and no aroma was detected from either. Achievement of blindness was validated before the trial in a group of 15 volunteers.

### Statistical analysis

Sample size was calculated using G*Power 3.1. The primary endpoint decrease of FSS score considered the four repeated measurements during one year of treatment, a size effect of 25 % as shown in a previous study [16], a 5 % error (alpha) and a potency of 80 %, with two groups and a correlation between the repeated measurements of 0.3. This resulted in a sample size of 24 patients.

The distribution probability of the scores was analysed with the Shapiro–Wilk test; evaluation of the normality of the data was performed using Graph Pad PRISM v6. In order to assess the effect of *A. paniculata* on the FSS and EDSS scores, a generalised linear mixed model (GLMM) analysis was performed. Treatment (*A. paniculata* and placebo), age (in years), sex (male, female) and time (four evaluations every three months) were considered to be fixed effects and patients were included as random effect. The GLMM analysis was done using R software [33].

## Results

**Fig. 1:**
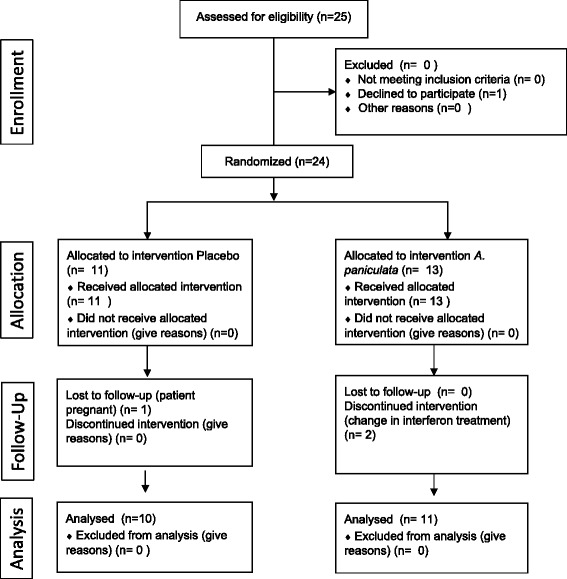
Flow chart of the 12-month double-blind placebo-controlled pilot study

The flow chart of the study is depicted in Fig. 1. Demographic parameters of the two groups included in the study are shown in Table 1.

In the placebo group, one patient did not complete the follow-up period and was excluded in the analysis due to pregnancy. In the *A. paniculata* group, two patients did not complete the follow-up period due to a change in the interferon treatment, which had been recommended by the study neurologists. Placebo 6/11 and *A. paniculata* 9/13 were fatigued according to the cut-off of 4.

### Effect of *A. paniculata* on relapse rate

No relapses occurred in the *A. paniculata* or placebo group during the 12-month protocol.

### Effect of *A. paniculata* on FSS score

**Fig. 2:**
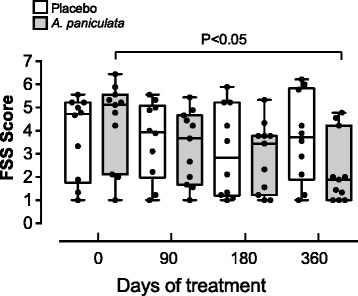
FSS score is reduced in RRMS patients treated with *A. paniculata* compared to placebo during one year. Each point represents an individual score of patients measured at 0, 90, 180 and 360 days. A box-and-whisker plot with the minimum, 25th percentile, median, 75th percentile, and maximum values are depicted

Overall, a reduction in FSS score was detected across time. No differences were detected between placebo and treatment; however, an interaction was detected between time and treatment, recording a reduction in FSS score when comparing the initial score (time 0) in the placebo group with the FSS score induced by *A. paniculata* after one year of treatment. The mean decrease in FSS score was 1.6, and ranged from 1.03 to 2.21 in a period of one year. In contrast, for the placebo group, the mean decrease in FSS score was 0.43 and ranged from 0.16 to 0.73 in a period of one year. Patients treated with *A. paniculata* showed a significant reduction in the FSS score compared to the placebo, equivalent to a 44 % reduction at 12 months (Fig. 2).

### Effect of *A. paniculata* on EDSS score

**Fig. 3:**
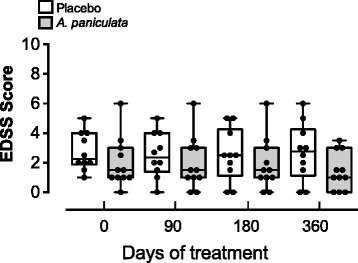
EDSS scores in RRMS patients treated with *A. paniculata* and placebo during one year. Each point represents an individual score of patients measured at 0, 90, 180 and 360 days. A box-and-whisker plot with the minimum, 25th percentile, median, 75th percentile, and maximum values are depicted

The EDSS score during the total period of treatment was similar for the *A. paniculata* and the placebo groups, and no significant differences were observed (Fig. 3). The GLMM analysis revealed that the *A. paniculata* group did not show a significant reduction of EDSS score compared to time 0. We found no differences within the placebo group.

### Effect of *A. paniculata* in magnetic resonance imaging (MRI)

Four patients within the placebo group showed new T2 lesions (mean 1; range 0–1), and new gadolinium-enhancing lesions (mean 1; range 0–1) at 12 months, compared to baseline MRI. In the *A. paniculata group*, only one patient showed one new T2 and one gadolinium-enhancing lesion.

### Safety

Only one patient in the *A. paniculata* group presented a mild and transient skin rash, which was alleviated with anti-histamine treatment for three weeks. The formulation was well tolerated and no changes in clinical parameters were observed.

## Discussion

In this placebo-controlled double-blind pilot study, we observed that *A. paniculata* treatment showed a significant reduction of 44 % in the FSS score compared to placebo at 12 months, and this effect seemed to be time dependent. As a symptom that highly impacts quality of life with high emotional, social and employment costs, the reduction of fatigue by *A. paniculata* treatment in MS was considered to be clinically meaningful. No effect of *A. paniculata* treatment on EDSS score was observed. Since EDSS does not include any aspects of fatigue, this result was expected.

Several pharmacological treatments available for fatigue in people with MS have failed to show clinical efficacy. A recently published comprehensive meta-analysis included seven clinical trials evaluating different medications used for the management of fatigue in MS patients. The authors found weak and inconclusive beneficial effects of pharmacological intervention limited to amantadine and Modafinil for MS-related fatigue, with small and non-significant pooled effect sizes, and a relatively narrow 95 % CI (pooled effect sizes = 0.07, 95 % CI: −0.22 to −0.37, *p* = 0.63) [34]. A previous systematic review that included the effects of amantadine and pemoline on MS fatigue also reported similar results [35]. Other comprehensive systematic reviews assessing the efficacy of 10 different studies on non-specific fatigue in palliative care investigating amantadine (*n* = 6), pemoline and Modafinil in MS patients [36] showed mixed results with weak and inconclusive data. Amantadine was found to demonstrate some improvement in fatigue in MS patients (meta-analysis of three-studies; standard mean difference compared to placebo, OR 1.68). Both pemoline and Modafinil failed to demonstrate a significant effect for management of fatigue in MS patients [15, 36]. In a randomised double-blind crossover clinical trial, patients treated with amantadine had their mean (SD) FSS decrease from 4.8 (1.4) to 4.0 (1.4) (P < 0 · 001), while patients treated with aspirin had their mean (SD) FSS decrease from 4.6 (1.4) to 3.5 (1.5) (*P* < 0 · 001), suggesting that both aspirin and amantadine significantly reduce MS-related fatigue [37]. The discrepancy of results could be related to the presence of confounders. A relationship has been proposed between MS-related fatigue and sleep disorders; moreover, treatment of underlying sleep disorder has led to an improvement of MS-related fatigue, and therefore should be considered as a relevant confounder [7].

Recently, alfacalcidol, a vitamin D analogue, has been shown to be able to decrease the mean FSS score compared to placebo (−41.6 % vs.−27.4 %, *p* = 0.007, respectively) and improve quality of life in MS patients [15].

Our results suggest that treatment with *A. paniculata* could be an effective pharmacological intervention in the mitigation of fatigue in RRMS patients treated with interferon beta. The mechanism of action by which *A. paniculata* exerts its effect in reducing fatigue is still unknown. Some evidence suggests that immunological factors, such as elevated levels of pro-inflammatory cytokines, may contribute to subjective fatigue in MS patients [38]. The main active compound of *A. paniculata*, andrographolide, can exert anti-inflammatory effects, [21, 39, 40] reducing pro-inflammatory cytokines, e.g., IFNγ [20, 41]. The assessment of cytokine profiles in MS patients with fatigue treated with *A. paniculata* should be considered.

Moreover, a direct effect of andrographolide in the CNS in the control of fatigue cannot be disregarded. Andrographolide is an apolar compound of low molecular weight that crosses the blood–brain barrier [42] and exerts neuroprotective effects [25].

The present study has limitations. For example, there was a small sample size, which was in part fine-tuned by the fact that we used a longitudinal study with repetitive measurements in time. However, in order to demonstrate the potential effect of *A. paniculata* on fatigue, a new study with a larger number of patients for a longer period of time (two years), the assessment of sleep disorders and the use of an additional fatigue scale differentiating between physical and cognitive fatigue, e.g., Fatigue Scale for Motor and Cognitive Functions (FSMC) [43], should also be considered.

## Conclusion

*A. paniculata* treatment shows a reduction in fatigue at one year in patients with RRMS receiving interferon beta in comparison to placebo and only interferon beta treatment. *A. paniculata* was safe and well tolerated, and no changes in clinical parameters were observed. Our sample size was limited, and we failed to achieve the necessary number of recruited patients for some parameters, specifically for the EDSS score. Indeed, a larger study including different scales and patient-oriented outcomes will be required to better determine the effect of *A. paniculata* on MS-related fatigue in patients with RRMS treated with interferon.

## Abbreviations

CNS: central nervous system
DC: dendritic cells
EAE: experimental autoimmune encephalomyelitis
EDSS: Expanded Disability Status Scale
FSS: Fatigue Severity Scores
HPLC: high-performance liquid chromatography
IFNγ: interferon gamma
IND: Investigational New Drug
IPH: Public Health of Chile
MRI: magnetic resonance imaging
MS: multiple sclerosis
RRMS: relapsing remitting multiple sclerosis

## References

[1] Hohlfeld R (2009). Multiple sclerosis: human model for EAE?. Eur J Immunol.

[2] Khan F, Amatya B, Galea M (2014). Management of fatigue in persons with multiple sclerosis. Front Neurol.

[3] Bakshi R (2003). Fatigue associated with multiple sclerosis: diagnosis, impact and management. Mult Scler.

[4] Lange R, Volkmer M, Heesen C, Liepert J (2009). Modafinil effects in multiple sclerosis patients with fatigue. J Neurol.

[5] Weinges-Evers N, Brandt AU, Bock M, Pfueller CF, Dorr J, Bellmann-Strobl J, Scherer P, Urbanek C, Boers C, Ohlraun S (2010). Correlation of self-assessed fatigue and alertness in multiple sclerosis. Mult Scler.

[6] Marrie RA, Reider N, Cohen J, Trojano M, Sorensen PS, Cutter G, Reingold S, Stuve O (2015). A systematic review of the incidence and prevalence of sleep disorders and seizure disorders in multiple sclerosis. Mult Scler.

[7] Veauthier C, Paul F (2014). Sleep disorders in multiple sclerosis and their relationship to fatigue. Sleep Med.

[8] Rocca MA, Meani A, Riccitelli GC, Colombo B, Rodegher M, Falini A, Comi G, Filippi M. Abnormal adaptation over time of motor network recruitment in multiple sclerosis patients with fatigue. Mult Scler*.* 2015:doi: 10.1177/1352458515614407. 10.1177/1352458515614407 26493126

[9] Calabrese M, Rinaldi F, Grossi P, Mattisi I, Bernardi V, Favaretto A, Perini P, Gallo P (2010). Basal ganglia and frontal/parietal cortical atrophy is associated with fatigue in relapsing-remitting multiple sclerosis. Mult Scler J.

[10] Sepulcre J, Masdeu JC, Goni J, Arrondo G, de Mendizabal NV, Bejarano B, Villoslada P (2009). Fatigue in multiple sclerosis is associated with the disruption of frontal and parietal pathways. Mult Scler J.

[11] Tartaglia MC, Narayanan S, Francis SJ, Santos AC, De Stefano N, Lapierre Y, Arnold DL (2004). The relationship between diffuse axonal damage and fatigue in multiple sclerosis. Arch Neurol.

[12] Filippi M, Rocca MA, Colombo B, Falini A, Codella M, Scotti G, Comi G (2002). Functional magnetic resonance imaging correlates of fatigue in multiple sclerosis. Neuroimage.

[13] Gottschalk M, Kumpfel T, Flachenecker P, Uhr M, Trenkwalder C, Holsboer F, Weber F (2005). Fatigue and regulation of the hypothalamo-pituitary-adrenal axis in multiple sclerosis. Arch Neurol.

[14] Flachenecker P, Bihler I, Weber F, Gottschalk M, Toyka KV, Rieckmann P (2004). Cytokine mRNA expression in patients with multiple sclerosis and fatigue. Mult Scler J.

[15] Achiron A, Givon U, Magalashvili D, Dolev M, Zaltzman SL, Kalron A, Stern Y, Mazor Z, Ladkani D, Barak Y (2015). Effect of Alfacalcidol on multiple sclerosis-related fatigue: A randomized, double-blind placebo-controlled study. Mult Scler J.

[16] Burgos RA, Hancke JL, Bertoglio JC, Aguirre V, Arriagada S, Calvo M, Caceres DD (2009). Efficacy of an Andrographis paniculata composition for the relief of rheumatoid arthritis symptoms: a prospective randomized placebo-controlled trial. Clin Rheumatol.

[17] Caceres DD, Hancke JL, Burgos RA, Sandberg F, Wikman GK (1999). Use of visual analogue scale measurements (VAS) to asses the effectiveness of standardized Andrographis paniculata extract SHA-10 in reducing the symptoms of common cold. A randomized double blind-placebo study. Phytomedicine.

[18] Caceres DD, Hancke JL, Burgos RA, Wikman GK (1997). Prevention of common colds with Andrographis paniculata dried extract. A pilot double blind trial. Phytomedicine.

[19] Sandborn WJ, Targan SR, Byers VS, Rutty DA, Mu H, Zhang X, Tang T (2013). Andrographis paniculata Extract (HMPL-004) for Active Ulcerative Colitis. Amer J Gastroenterol.

[20] Carretta MD, Alarcon P, Jara E, Solis L, Hancke JL, Concha II, Hidalgo MA, Burgos RA (2009). Andrographolide reduces IL-2 production in T-cells by interfering with NFAT and MAPK activation. Eur J Pharmacol.

[21] Hidalgo MA, Romero A, Figueroa J, Cortes P, Concha II, Hancke JL, Burgos RA (2005). Andrographolide interferes with binding of nuclear factor-kappaB to DNA in HL-60-derived neutrophilic cells. Br J Pharmacol.

[22] Iruretagoyena MI, Tobar JA, Gonzalez PA, Sepulveda SE, Figueroa CA, Burgos RA, Hancke JL, Kalergis AM (2005). Andrographolide interferes with T cell activation and reduces experimental autoimmune encephalomyelitis in the mouse. J Pharmacol Exp Ther.

[23] Wang TG, Liu B, Zhang W, Wilson B, Hong JS (2004). Andrographolide reduces inflammation-mediated dopaminergic neurodegeneration in mesencephalic neuron-glia cultures by inhibiting microglial activation. J Pharmacol Exp Ther.

[24] Das S, Gautam N, Dey SK, Maiti T, Roy S (2009). Oxidative stress in the brain of nicotine-induced toxicity: protective role of Andrographis paniculata Nees and vitamin E. Appl Physiol Nutr Me.

[25] Chan SJ, Wong WSF, Wong PTH, Bian JS (2010). Neuroprotective effects of andrographolide in a rat model of permanent cerebral ischaemia. Br J Pharmacol.

[26] Cabrera D, Pizarro M, Solis N, Torres J, Brandan E, Arrese M (2013). Effects of andrographolide in experimental non-alcoholic steatohepatitis. Hepatology.

[27] Polman CH, Reingold SC, Banwell B, Clanet M, Cohen JA, Filippi M, Fujihara K, Havrdova E, Hutchinson M, Kappos L (2011). Diagnostic criteria for multiple sclerosis: 2010 revisions to the McDonald criteria. Ann Neurol.

[28] Schulz KF, Altman DG, Moher D, Group C (2010). CONSORT 2010 Statement: updated guidelines for reporting parallel group randomised trials. BMC Med.

[29] Panossian A, Hovhannisyan A, Mamikonyan G, Abrahamian H, Hambardzumyan E, Gabrielian E, Goukasova G, Wikman G, Wagner H (2000). Pharmacokinetic and oral bioavailability of andrographolide from Andrographis paniculata fixed combination Kan Jang in rats and human. Phytomedicine.

[30] Burgos RA, Caballero EE, Sanchez NS, Schroeder RA, Wikman GK, Hancke JL (1997). Testicular toxicity assessment of Andrographis paniculata dried extract in rats. J Ethnopharmacol.

[31] Krupp LB, LaRocca NG, Muir-Nash J, Steinberg AD (1989). The fatigue severity scale. Application to patients with multiple sclerosis and systemic lupus erythematosus. Arch Neurol.

[32] Perez S, Hervada X, Naveira G, Silva L, Fariñas H, Vázquez E, Bacallao J, Mújica O (2010). The epidat program. Rev Panam Salud Publica.

[33] R-Development-Core-Team. R: A Language and Environment for Statistical Computing. Vienna, Austria: the R Foundation for Statistical Computing. Available online at https://www.r-project.org*.* Accessed 20 Dec 2015.

[34] Asano M, Finlayson ML (2014). Meta-analysis of three different types of fatigue management interventions for people with multiple sclerosis: exercise, education, and medication. Mult Scler Int.

[35] Branas P, Jordan R, Fry-Smith A, Burls A, Hyde C (2000). Treatments for fatigue in multiple sclerosis: a rapid and systematic review. Health Technol Assess.

[36] Peuckmann V, Elsner F, Krumm N, Trottenberg P, Radbruch L (2010). Pharmacological treatments for fatigue associated with palliative care. Cochrane Database Syst Rev.

[37] Shaygannejad V, Janghorbani M, Ashtari F, Zakeri H (2012). Comparison of the effect of aspirin and amantadine for the treatment of fatigue in multiple sclerosis: a randomized, blinded, crossover study. Neurol Res.

[38] Hanken K, Eling P, Hildebrandt H (2014). The representation of inflammatory signals in the brain - a model for subjective fatigue in multiple sclerosis. Front Neurol.

[39] Lee KC, Chang HH, Chung YH, Lee TY (2011). Andrographolide acts as an anti-inflammatory agent in LPS-stimulated RAW264.7 macrophages by inhibiting STAT3-mediated suppression of the NF-kappaB pathway. J Ethnopharmacol.

[40] Lu CY, Yang YC, Li CC, Liu KL, Lii CK, Chen HW (2014). Andrographolide inhibits TNFalpha-induced ICAM-1 expression via suppression of NADPH oxidase activation and induction of HO-1 and GCLM expression through the PI3K/Akt/Nrf2 and PI3K/Akt/AP-1 pathways in human endothelial cells. Biochem Pharmacol.

[41] Burgos RA, Seguel K, Perez M, Meneses A, Ortega M, Guarda MI, Loaiza A, Hancke JL (2005). Andrographolide inhibits IFN-gamma and IL-2 cytokine production and protects against cell apoptosis. Planta Med.

[42] Lu W (1995). Prospect for study on treatment of AIDS with traditional Chinese medicine. J Tradit Chin Med.

[43] Penner IK, Raselli C, Stocklin M, Opwis K, Kappos L, Calabrese P (2009). The Fatigue Scale for Motor and Cognitive Functions (FSMC): validation of a new instrument to assess multiple sclerosis-related fatigue. Mult Scler.

